# Attention Performance Measured by Attention Network Test Is Correlated with Global and Regional Efficiency of Structural Brain Networks

**DOI:** 10.3389/fnbeh.2016.00194

**Published:** 2016-10-10

**Authors:** Min Xiao, Haitao Ge, Budhachandra S. Khundrakpam, Junhai Xu, Gleb Bezgin, Yuan Leng, Lu Zhao, Yuchun Tang, Xinting Ge, Seun Jeon, Wenjian Xu, Alan C. Evans, Shuwei Liu

**Affiliations:** ^1^Research Center for Sectional Imaging Anatomy, Shandong Provincial Key Laboratory of Mental Disorders, School of Medicine, Shandong UniversityJinan, China; ^2^Montreal Neurological Institute, McGill University, MontrealQC, Canada; ^3^Laboratory of Neuroimaging, Institute of Neuroimaging and Informatics, Keck School of Medicine, University of Southern California, Los AngelesCA, USA; ^4^Department of Radiology, Affiliated Hospital of Qingdao UniversityQingdao, China

**Keywords:** attention, diffusion tensor imaging, structural brain network, graph analysis, attention network test

## Abstract

Functional neuroimaging studies have indicated the involvement of separate brain areas in three distinct attention systems: alerting, orienting, and executive control (EC). However, the structural correlates underlying attention remains unexplored. Here, we utilized graph theory to examine the neuroanatomical substrates of the three attention systems measured by attention network test (ANT) in 65 healthy subjects. White matter connectivity, assessed with diffusion tensor imaging deterministic tractography was modeled as a structural network comprising 90 nodes defined by the automated anatomical labeling (AAL) template. Linear regression analyses were conducted to explore the relationship between topological parameters and the three attentional effects. We found a significant positive correlation between EC function and global efficiency of the whole brain network. At the regional level, node-specific correlations were discovered between regional efficiency and all three ANT components, including dorsolateral superior frontal gyrus, thalamus and parahippocampal gyrus for EC, thalamus and inferior parietal gyrus for alerting, and paracentral lobule and inferior occipital gyrus for orienting. Our findings highlight the fundamental architecture of interregional structural connectivity involved in attention and could provide new insights into the anatomical basis underlying human behavior.

## Introduction

Attention is the behavioral and cognitive process of the preparedness for and selection of certain physical environments or some mental aspects stored in memory (Raz and Buhle, 2006). Although many competing theories have proposed different potential components of attention, emerging brain imaging studies have consistently supported the idea that there are three distinct key subsystems of attention, namely alerting, orienting, and executive control (EC) (Fan et al., 2005; Posner, 2008; Niogi et al., 2010). Briefly, alerting refers to the ability to achieve and maintain a state of high sensitivity; orienting is defined as the ability to select information from sensory stimuli; and EC is involved with resolving cognitively incongruent stimuli (Posner, 2008). Functional neuroimaging studies have demonstrated that these three attentional functions are mediated by anatomically separate neural networks (Fan et al., 2005). The alerting component involves activation of frontal, parietal, and thalamic regions (Coull et al., 1996; Beane and Marrocco, 2004). The orienting network depends largely on superior frontal and parietal areas, temporal parietal junction, and precentral and postcentral gyrus (Corbetta and Shulman, 2002; Fan et al., 2005). The EC system has important involvement of frontal areas including dorsolateral and medial prefrontal cortex, thalamus and anterior cingulate cortex (Matsumoto and Tanaka, 2004; Fan et al., 2005; Aron et al., 2014).

The attention network test (ANT), designed by Fan et al. (2005), provided a means for differentiating these three independent attentional components in a single integrated task. The ANT has been widely used to quantify the attentional performance in both healthy individuals (Westlye et al., 2011; Joseph et al., 2015) and neuropsychiatric patients with attentional deficits, including schizophrenia (Breton et al., 2011; Spagna et al., 2015), attention deficit hyperactivity disorder (ADHD) (Kratz et al., 2011; Mogg et al., 2015), and autism (Keehn et al., 2010; Fan et al., 2012). However, aforementioned neuroimaging studies mainly focused on the roles played by each brain region of the three attentional networks. With such univariate approaches, interactions among brain regions within these networks were not evaluated. Network-based measures, on the other hand, could be more sensitive to brain changes which are less evident in gross structure, as each region’s integration is considered into a global unit, rather than an independent entity. Large-scale network analysis using graph theoretical approaches offers a new way to investigate the structural and functional integration underlying human cognitive function and to discover the potential biological mechanism responsible for brain dynamics (Zhang et al., 2012; Sporns, 2013).

Using resting-state fMRI, a recent study (Xu et al., 2015) applied graph theory to examine the relationship between functional network characteristics and behavioral measures of attention as provided by ANT, and found that the performances of the three attentional components were associated with specific nodal regional efficiency. These regions [such as the left superior parietal gyrus and thalamus for the alerting effect, the left superior frontal gyrus (SFG) and fusiform gyrus for the orienting system, and the right inferior frontal gyrus and anterior cingulated gyrus for the EC component] were largely consistent with the findings in previous task-related activation studies. Using such graph theoretical approaches, another resting-state fMRI study (Markett et al., 2014) discovered that a higher global connectivity could be disadvantageous for the alerting performance. The authors also showed that the degree centrality of the right ventral intraparietal sulcus showed a negative association with EC effect. Although these studies revealed the functional network characteristics of attention, the anatomical substrates underlying attentional performance remains unclear. Diffusion tractography methods using diffusion tensor imaging (DTI) provide an opportunity for investigating the brain anatomical connectivity *in vivo*, which enables us to reveal the basic topological features of the large-scale structural network across the entire brain. In previous neuroimaging investigations, structural brain networks obtained from tractography have been successfully applied to healthy subjects (Gong et al., 2009; Sun et al., 2015), as well as diseased population, such as ADHD (Cao et al., 2013; Sidlauskaite et al., 2015), Schizophrenia (van den Heuvel et al., 2010) and Alzheimer’s Disease (AD) (Daianu et al., 2015).

Therefore, the present study aimed to utilize diffusion tractography and graph theoretical measures on 65 healthy subjects to establish the neuroanatomical correlates of the three ANT networks. We calculated both global and nodal topological properties of structural brain networks for each subject. Then we assessed the linear regression analysis between the topological characteristics and attentional performance measured by ANT. On the basis of previous neuroimaging works (Fan et al., 2005; Posner, 2008), we hypothesized association of the executive function with global network topology. We also hypothesized that the behavioral performance of the three attentional sub-networks will display correlation with different brain areas. Our results could provide useful information of fundamental architecture of interregional structural connectivity involved in attention and provide new insights into the anatomical basis underlying attentional deficits in both neuropsychiatric and neurological disorders.

## Materials and Methods

### Subjects

A total of 65 healthy subjects (29 males, average age: 17.17 ± 1.59 years old) were recruited in this study. All subjects were Chinese native speakers, with no history of neurological or psychological illness and no abnormal findings in conventional brain MRI, corresponding to Diagnostic and Statistical Manual of Mental Disorders (DSM-IV). All subjects were right-handed measured with the Edinburgh handedness inventory (Oldfield, 1971). The study was approved by the Human Research Ethics Committee of Shandong University School of Medicine. Written informed consents were obtained from all the participants as well as their parents.

### Behavioral Task

A version of ANT devised by Fan et al. (2005) was used as a cognitive task to examine the efficiency of the alerting, orienting, and EC networks involved in attention. Participants were instructed to press a button as accurately and quickly as possible to identify the direction of the target, which was a leftward or rightward arrow at the center and flanked on either side by two arrows in the same direction (congruent condition), or the opposite direction (incongruent condition). The target and flankers were presented until the participant made a response or 2000 ms elapsed. A cue (an asterisk) was presented for 200 ms before the target appeared. The task used three cue conditions: no cue (baseline), center cue (at the fixation for alerting), and spatial cue (at the target location for alerting and orienting).

Each subject performed six blocks in this experiment, each block lasting 5 min 42 s and consisting of 36 trials plus 2 buffer trials at the beginning. In each block, the six trial types (three cue conditions by two target conditions) were presented in a predetermined counterbalanced order. All subjects were trained before the formal experiment. Stimulus presentation and behavioral response collection were performed using E-Prime (Psychology Software Tools, Pittsburgh, PA, USA) on an experimental control computer.

### Behavioral Data Analysis

The total accuracy of each subject was calculated and those with high error rates (>20%) should be excluded from this study. The trials with incorrect responses or with response time (RT) longer than 1500 ms or shorter than 200 ms were also excluded to avoid the influence of the outliers. In addition, we need remove responses following erroneous ones to avoid post-error slowing effect. Since RTs were not normally distributed, we used median RT per condition as raw scores (Adolfsdottir et al., 2008). The accuracy for each of the six trial types was also calculated. Finally, instead of the conventional subtraction measure (Fan et al., 2005, 2007), we used ratio scores of alerting, orienting, and EC to define the effects of three attention networks. The formulas were as follows:

$$\text{Alerting effect=}({RT}_{no\,\;cue}-{RT}_{center\,\;cue})/{RT}_{center\,\;cue}$$

$$\text{Orient effect=}({RT}_{center\,\;cue}-{RT}_{spatial\,\;cue})/{RT}_{spatial\,\;cue}$$

$$\text{EC effect = }({RT}_{incongruent}-{RT}_{congruent})/{RT}_{congruent}$$

The executive attention effect was calculated by subtracting the mean RT of congruent conditions from the mean RT of incongruent conditions; thus, a higher EC score reflected a relatively poorer executive function.

### MRI Data Acquisition and DTI Preprocessing

MR imaging data were acquired using a 3.0 Tesla GE Signa scanner (General Electric Medical Systems, Milwaukee, WI, USA) with a standard eight-channel head coil. DTI images were obtained using spin-echo, single shot echo planar imaging sequence [TR/TE = 14000/75.1 ms, acquisition matrix = 96 × 96, field of view (FOV) = 250 mm, slice thickness = 2.6 mm, no gap]. The DTI scheme included 30 non-linear diffusion gradients directions with *b* = 1000 s/mm^2^ and 3 non-diffusion-weighted images (*b* = 0 s/mm^2^). Array spatial sensitivity encoding technique (ASSET) was adapted with an acceleration factor of 2 to reduce acquisition time with less image distortion from susceptibility artifacts (Yu et al., 2008). For each subject 56 axial slices were collected and the diffusion sequence was repeated twice to increase signal-to-noise ratio (SNR). At the end of the DTI scans, a three-dimensional volume spoiled gradient-echo (SPGR) pulse sequence with 174 slices (TR = 6.5 ms, TE = 2.0 ms, matrix = 256 × 256, FOV = 256 mm, FA = 15°, slice thickness = 1.0 mm, no gap) was used to acquire the structural images for anatomical reference.

The DTI data were pre-processed using FSL (University of Oxford, UK) to correct for eddy currents and head motion. After two acquisitions being averaged, the averaged images were masked to remove the skull and non-brain tissue using the FSL Brain Extraction Tool (BET) (Smith, 2002). Afterward, the fractional anisotropy (FA) was calculated using the diffusion tensor analysis toolkit (FDT; Smith et al., 2004).

### Construction of Structural Brian Networks

**FIGURE 1:**
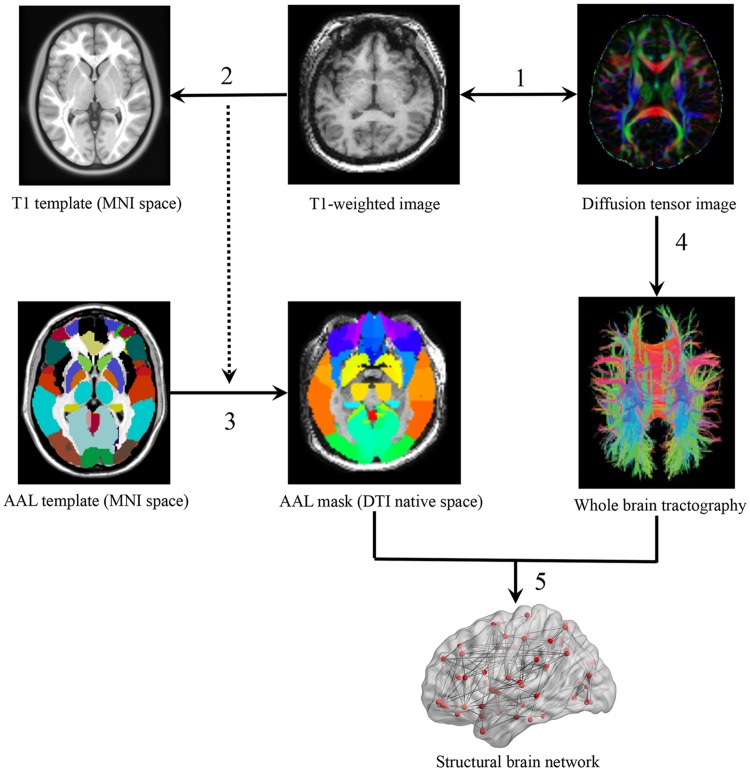
**A flowchart of WM network construction.** (1) Rigid coregistration from individual T1-weighted image to diffusion tensor imaging (DTI) native space (DTI color-coded map represents the directions of first eigenvectors: red, left to right; green, anterior to posterior; blue, inferior to superior). (2) Non-linear registration from T1-weighted images to ICBM152 T1 template in Montreal Neurological Institute space, resulting in a non-linear transformation (Γ). (3) Application of the inverse transformation (Γ-1) to the AAL atlas, resulting in corresponding AAL mask in each individual’s DTI native space. (4) Reconstruction of whole-brain tractography using fiber assignment by continuous tracking (FACT) algorithm. (5) Network constructions by determining the white matter connections for each pair of AAL areas.

The whole cerebral cortex was parcellated into 90 areas using the automated anatomical labeling (AAL) template (Tzourio-Mazoyer et al., 2002) where each region represented a WM network node (Bullmore and Sporns, 2009). In the parcellation process, each individual T1 image was linearly coregistered to the *b*_0_ image in the native space and then non-linearly mapped to the International Consortium for Brain Mapping 152 (ICBM 152) T1 template in the Montreal Neurological Institute (MNI) space, resulting in a non-linear transformation (Γ). Finally, one subject-specific AAL mask in the DTI native space was generated, using the inverse transformation (Γ^-1^) to the AAL template in the MNI space (**Figure 1**).

Network edges were defined as the interregional anatomical connections determined using DTI deterministic tractography. Fibers with two end-points located in their respective masks were considered as edges to link corresponding pairs of nodes. The whole-brain fiber tracking was performed via Fiber Assignment by Continuous Tracking (FACT) algorithm (Mori et al., 1999), with the FA threshold of 0.1 and tracking turning angular threshold of 30° between two connections. PANDA (Cui et al., 2013) was used to generate a FA-weighted matrix M(90*90) with cell M(i,j) representing the averaged FA of linking fibers between node *i* and node *j*. All cells of *M* that exceeded the threshold of 0.1 were set to 1 and other cells that did not exceed 0.1 were set to 0. This resulted in a fully connected binary matrix B (van den Heuvel et al., 2009; Xu et al., 2015).

### Graph Theory Analysis

With the binary connectivity matrices (B), topological properties of structural brain networks were obtained using Brainnetome fMRI Toolkit^1^, including small-worldness, global efficiency, local efficiency and regional efficiency.

#### Small-Worldness

A real network would be counted as small-world network if it meets the following criteria (Watts and Strogatz, 1998): $\gamma =C_{p}^{real}/C_{p}^{rand}>1,\;\lambda =L_{p}^{real}/L_{p}^{rand}\approx 1,where\,\;C_{p}^{rand}\,\;and\,\;L_{p}^{rand}$ represents the mean clustering coefficient and characteristic path length of 100 random networks which have the same number of nodes, edges and degree distribution with the real network (Maslov and Sneppen, 2002; Sporns and Zwi, 2004). *C* is defined as the average clustering-coefficient over all nodes: $C=\frac{1}{N}\Sigma_{i\epsilon G}\,\;C_{i}$, where *C*_i_ of node *i* is defined as the ratio of the number of existing edges between the neighbors of node *i* and the numbers of all possible edges between its neighbors: $C_{i}=\frac{E_{i}}{K_{i}(K_{i}-1)/2}$, with *E*_i_ being the number of edges of the sub-graph of neighbors of node *i* and *K*_i_ being the number of edges of node *i* (Watts and Strogatz, 1998; Latora and Marchiori, 2001). The characteristic shortest path length *L* is defined as the averaged *L*_i_ over all nodes: $L=\frac{1}{N}\Sigma_{i\epsilon G}\,\;L_{i}$, where *L*_i_ representing the averaged minimal number of connections which link node *i* to every other nodes in the graph: $L_{i}=\frac{1}{N-1}\Sigma_{i\neq j\epsilon G}\,\;\min\left\{L_{i,j}\right\}$, with *L*_i,j_ being the number of edges between node *i* and node *j* and min{*L*_i,j_} being the shortest path length among *L*_i,j_ (Latora and Marchiori, 2001; Achard et al., 2006).

#### Regional and Global Efficiency

The regional efficiency *E*_regional_ for a given node i is a measure of how connected the node *i* is to all other nodes in the network G and is defined as: $E_{regional}=\frac{1}{N-1}\,\;\Sigma_{i,j\epsilon G,i\neq j}\,\;\frac{1}{l_{i,j}}$. Here, *l*_i,j_ is the minimum path length between node *i* and *j*. The global efficiency of a network *G* is defined as: $E_{glob}=\frac{1}{N(N-1)}\,\;\Sigma_{i,j\epsilon G,i\neq j}\,\;\frac{1}{l_{i,j}}$, which is the inverse of the harmonic mean of the shortest path lengths of each pair of nodes, quantifying parallel information transfer throughout the entire network, i.e., how integrated the network is (Latora and Marchiori, 2001).

#### Local Efficiency

The local efficiency of node is defined as: E_i_loc_ = E_glob_(G_i_), which indicates how efficient the information transfer in *G*_i_ when the node *i* is eliminated (Achard and Bullmore, 2007). The local efficiency of a network is defined as: $E_{loc}=\frac{1}{N}\,\;\Sigma_{i\epsilon G}\,\;E_{i\_loc}$, which is the mean local efficiency over all nodes in the network.

### Statistical Analysis

SPSS (version 20, IBM Inc., Chicago, IL, USA) was used for statistical analyses. To explore the relationship between global brain network topology (the global efficiency, *E*_glob_, and the local efficiency, *E*_loc_) and behavioral performances of the three attentional components, Pearson’s linear correlations were separately performed on each network metric, using gender and age as covariates. We then apply Bonferroni correction for multiple testing to the results.

To identify specific brain regions involved in alerting, orienting and EC, multiple linear regression analyses for nodal efficiency of all 90 cortical regions and three attentional effects were performed with SPSS. As the problem of multicollinearity occurred due to correlation between the predictors, we first used multicollinearity diagnostics to assess the independence of parameters and to test whether two or more variables were highly correlated. Then, we apply ninety blocks of independent variables in a step-wise fashion. Statistical threshold at the level of *p* < 0.05 was considered significant.

## Results

### ANT Components

The average accuracy of ANT performance was high (0.97 ± 0.02) and no one was excluded from the study, indicating that all participants understood the instructions and were able to perform the task reliably.

**Table 1** showed the three effect ratio scores of ANT components and their correlations. Independent-sample *t*-tests revealed no significant gender differences in alerting, orienting and EC effects. Only a significant correlation (*r* = -0.35, *p* = 0.005) between the alerting and orienting performance was found, after controlling for gender and age.

**Table 1:** Three attentional sub-network effects for females, males (Means ± SD) and their correlation coefficients.

	Sample size	Alerting (%)	Orienting (%)	EC (%)
Male	29	5.61 ± 2.69	8.77 ± 3.36	16.40 ± 3.73
Female	36	6.20 ± 3.76	9.25 ± 4.5	14.67 ± 4.28
*t*(*p*)		-0.71 (0.48)	-0.50 (0.62)	1.72 (0.09)
Orienting	65	-0.35 (0.005^∗^)	1	
EC	65	-0.04 (0.76)	-0.21 (0.10)	1


*The attention network test (ANT) effects are shown in percent relative to the baseline condition. *t* denotes the *t*-value obtained from independent sample *t*-tests. ^∗^Correlation is significant at the 0.05 level.*

### Relationship between ANT Effects and Global Topological Properties

**FIGURE 2:**
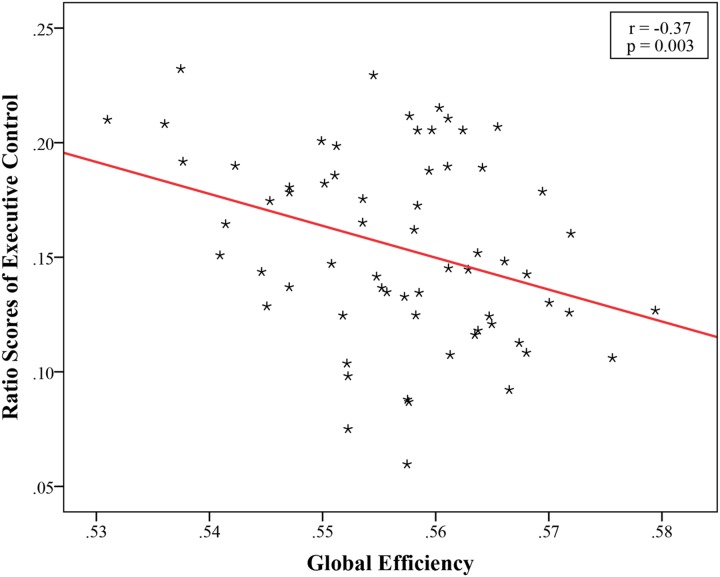
**Significant negative correlation between global efficiency and ratio scores of Executive Control, EC (*r* = -0.37, *p* = 0.003, Bonferroni corrected)**.

The structural brain networks were constructed for each subject, with 90 nodes and 4005 possible edges and the global topological properties of structural networks spanning the entire brain were then examined. The constructed structural brain networks showed small-world characteristics expressed as γ > 1 and λ ≈ 1 (σ = 1.76 ± 0.10, γ = 1.94 ± 0.12, λ = 1.10 ± 0.01). We found a significant correlation (*r* = -0.37, *p* = 0.003, Bonferroni corrected) between the EC effect and global efficiency (**Figure 2**; **Table 2**). No significant associations were observed between global network properties and ratio scores of alerting and orienting.

**Table 2:** Linear correlations between the ANT effects and global properties of structural brain networks (age and gender partialled out, Bonferroni corrected).

	Global efficiency	Local efficiency
Alerting	0.09 (0.51)	0.07 (0.57)
Orienting	0.03 (0.83)	-0.10 (0.44)
EC	-0.37 (0.003^∗^)	-0.13 (0.32)


*^∗^Correlation is significant at the 0.05 level.*

### Relationship between ANT Effects and Region-Based Network Properties

To further identify which specific brain regions are related to any of the three attention variables, multiple linear regression analysis was performed between nodal characteristics and ratio scores of alerting, orienting and EC for individual brain networks. The correlations between these brain regions were low and no significant multicollinearity were found. Some regions were observed whose regional efficiency significantly correlated with the behavioral performances of three components of attention, as shown in **Tables 3**–**5**.

**Table 3:** Results of multiple linear regression for EC and regional efficiency of specific brain regions (Model summary: *R*^2^ = 0.648; *F* = 11.247; *P* = 0.000).

*Y*	*x*_i_	β_i_	*P*_i_	Lower bound (95% CI)	Upper bound (95% CI)
EC	Constant	1.473	0.000	1.101	1.845
	L Superior frontal gyrus ^a^	-0.538	0.000	-0.739	-0.338
	L Parahippocampal gyrus	0.292	0.021	0.046	0.537
	L Thalamus	0.268	0.032	0.024	0.512
	R Thalamus	-0.632	0.000	-0.887	-0.377
	R Supplementary motor area	0.216	0.038	0.012	0.420
	L Pallidum	-0.304	0.015	-0.548	-0.061
	R Pallidum	-0.363	0.005	-0.613	-0.113
	R Putamen	-0.525	0.000	-0.740	-0.310
	R Calcarine fissure	-0.595	0.000	-0.844	-0.347


**Y* = β_0_ + β_1_ × x_1_ + β_2_ × x_2_ + …+ β_i_ × x_i._^a^ dorsolateral; EC, executive control; L, Left; R, Right; CI, Confidence Interval.*

**Table 4:** Results of multiple linear regression for alerting and regional efficiency of specific brain regions (Model summary: *R*^2^ = 0.288; *F* = 6.072; *P* = 0.000).

*Y*	*x*_i_	β_i_	*P*_i_	Lower bound (95% CI)	Upper bound (95% CI)
Alerting	Constant	-0.383	0.001	-0.607	-0.160
	L Thalamus	0.402	0.001	0.163	0.641
	R Inferior Parietal Gyrus	0.250	0.036	0.017	0.483
	L Middle Temporal Gyrus	-0.252	0.015	-0.453	-0.050
	R Pallidum	0.426	0.002	0.166	0.685


**Y* = β_0_ + β_1_ × x_1_ + β_2_ × x_2_ + … + β_i_ × x_i._ L, Left; R, Right; CI, Confidence Interval.*

**Table 5:** Results of multiple linear regression for orienting and regional efficiency of specific brain regions (Model summary: *R*^2^ = 0.283; *F* = 8.009; *P* = 0.000).

*Y*	*x*_i_	β_i_	*P*_i_	Lower bound (95% CI)	Upper bound (95% CI)
Orienting	Constant	-0.058	0.594	-0.274	0.159
	R Paracentral Lobule	0.376	0.020	0.062	0.690
	R Inferior Occipital Gyrus	0.319	0.003	0.115	0.522
	L Temporal pole	-0.383	0.000	-0.583	-0.183


**Y* = β_0_ + β_1_ × x_1_ + β_2_ × x_2_ + … + β_i_ × x_i._ L, Left; R, Right; CI, Confidence Interval.*

**FIGURE 3:**
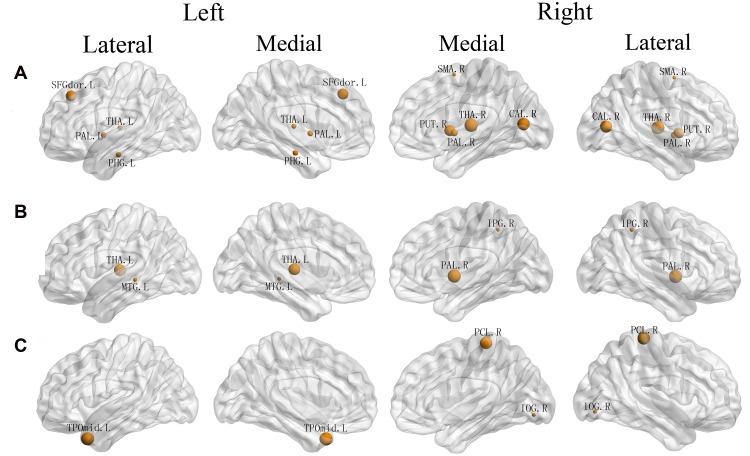
**Three-dimensional representations of the regions whose regional efficiency was significantly correlated with three attentional effect **(A**: EC, **B**: alerting, **C**: orienting).** Node sizes indicate their correlation coefficient with alerting. Nodal regions were mapped onto the cortical surface at the lateral and medial view. L, Left; R, Right; SFGdor, Superior frontal gyrus, dorsolateral; PHG, Parahippocampal gyrus; THA, Thalamus; SMA, Supplementary motor area; PAL, Lenticular nucleus, Pallidum; PUT, Lenticular nucleus, Putamen; CAL, Calcarine fissure and surrounding cortex; IPG, Inferior Parietal Gyrus; MTG, Middle Temporal Gyrus; PCL Paracentral Lobule; IOG, Inferior Occipital Gyrus; TPOmid, Temporal pole, middle temporal gyrus.

Significant correlations between EC component and regional efficiency of a set of brain areas (**Table 3**; **Figure 3A**) were discovered, including the left dorsolateral SFG (β = -0.538, *p* = 0.000), left parahippocampal gyrus (β = 0.292, *p* = 0.021), lateral thalamus (left: β = 0.268, *p* = 0.032; right: β = -0.632, *p* = 0.000), right supplementary motor area (β = 0.216, *p* = 0.038), lateral pallidum (left: β = -0.304, *p* = 0.015; right: β = -0.363, *p* = 0.005), right putamen (β = -0.525, *p* = 0.000) and right calcarine fissure (β = -0.595, *p* = 0.000).

As shown in **Table 4** and **Figure 3B**, we observed significant correlations between alerting and regional efficiency of the left thalamus (β = 0.402, *p* = 0.001), right inferior parietal gyrus (*r* = 0.250, *p* = 0.036), left middle temporal gyrus (β = -0.252, *p* = 0.015) and right lenticular nucleus, Pallidum (β = 0.462, *p* = 0.002). For the orienting effect, the significant associations (**Table 5**; **Figure 3C**) with the regional efficiency were found in the right paracentral lobule (β = 0.376, *p* = 0.020), right inferior occipital gyrus (β = 0.319, *p* = 0.003) and left temporal pole (middle temporal gyrus: β = -0.383, *p* = 0.000).

## Discussion

In the present study, graph theory was applied to structural brain networks constructed from DTI images to investigate correlations between global and regional topological characteristics and the three attentional effects within the ANT task. The EC component showed a significant correlation with the global efficiency of the entire structural brain networks. Furthermore, we found direct evidence supporting our hypothesis that region-based network properties significantly correlated with the three ANT systems. Nodal efficiencies of specific regions, including the dorsolateral SFG, thalamus and parahippocampal gyrus significantly correlated with EC; thalamus and inferior parietal gyrus with alerting; and paracentral lobule and inferior occipital gyrus correlated with orienting.

### Attentional Effects on Whole Brain Structural Network Properties

Graph theoretical analysis of structural and functional neuroimaging data have shown small-world architecture of human brain indicating high clustering of connections as well as shortest path length between brain regions, which in turn, supports the optimal balance between local specialization and global integration (Bullmore and Sporns, 2009). Consistent with these findings, the structural brain networks constructed from DTI data in this study also showed a small-world organization. Based on graph theory, recent neuroimaging studies have measured the relationships between brain network architecture and cognitive functions, like intellectual performance (van den Heuvel et al., 2009) and working memory (Gamboa et al., 2014). However, graph-theoretic studies of attention have mainly focused on functional brain networks, with few studies on structural brain networks from DTI data. Our study on structural brain networks suggested the analogous discoveries with previous studies on functional brain networks (Markett et al., 2014; Xu et al., 2015).

In this study, the global efficiency of WM networks showed a significant positive correlation with the EC performance. Global efficiency reflects the network’s capacity for parallel information transfer between brain regions via multiple parallel paths, and is believed to be the basis of integrated processing for cognitive functions. Higher scores on intelligence tests were found corresponding to a higher global efficiency of both WM networks (Li et al., 2009) and functional brain networks (van den Heuvel et al., 2009). Patients with hypertension (Li et al., 2015b), amnestic mild cognitive impairment (aMCI) (Shu et al., 2012) or traumatic brain injury (TBI) (Caeyenberghs et al., 2014) demonstrated a decreased global efficiency of the WM networks, resulting in decline of the executive function. Lo et al. (2010) found that AD patients with impairments in cognition and memory functions, exhibited a decreased global efficiency of the whole WM networks. Therefore, the executive function tends to rely on a more optimal configuration (small-worldness) of structural brain networks, and a limited capacity to integrate information across brain regions would result in a poor executive performance in cognitive deficits. Additionally, our results provided evidences that network measures could be an efficient way to quantify the structural brain system underlying cognitive functions.

### Attentional Effects on Region-Based Structural Network Properties

We examined the correlations not only at the whole brain network level, but also at the individual regional level. From this perspective, a multiple linear regression analysis was conducted in the regional efficiency of each brain region to investigate specific regions involved in the three ANT components.

#### Executive Control (EC)

In accordance with our assumption based on previous neuroimaging and lesion studies, we discovered significant correlations between EC effect and regional efficiency of left dorsolateral SFG. It is widely accepted that the dorsolateral prefrontal cortex is the core substrate of EC operations based on previous event-related fMRI and neurophysiological studies (Wagner et al., 2001; Fan et al., 2005). Also, dorsolateral frontal areas are key components in fronto-parietal control network, which is a resting state network known to be related to EC (Spreng et al., 2013). In addition, a previous structural study with a large sample of 342 healthy individuals aged 72–92 also revealed that better executive function significantly correlated with greater regional efficiency of left SFG, independently of age, sex, and years of education (Wen et al., 2011). More importantly, some diseases with cognitive deficits were found to be accompanied by disrupted morphology (Gianaros et al., 2006), metabolism (Noel et al., 2002), and functional connectivity (Li et al., 2015a) mainly in the superior frontal areas. From an anatomical perspective, our results provided direct evidence for the involvement of this areas in cognitive control processes.

Regional efficiency of the left parahippocampal gyrus was found significantly correlated with EC. This finding was supported by previous fMRI studies characterizing the functional role of parahippocampal gyrus associated with executive function (Kubler et al., 2006; Chan and Lavallee, 2015). Parahippocampal gyrus played important role in the default mode network, which is a known resting state network influenced EC (Spreng et al., 2009). Based on graph theory, a recent resting-state fMRI study also found EC was significantly correlated with regional efficiency of left parahippocampal gyrus (Xu et al., 2015). Additionally, patients with EC deficits, like SZ (Shenton et al., 2001), Parkinson’s disease (Burton et al., 2004) showed gray mater volume reduction in the left parahippocampal gyrus and major depressive disorder (MDD) (Peng et al., 2011) showed gray matter density reduction in this area. The parahippocampal gyrus is therefore considered to be not only involved in EC morphologically, but also an important hub transferring information with other regions.

We also found regional efficiency of thalamus, pallidum, putamen and supplementary motor area associated with EC. These regions are hubs for fronto-parietal control network, which is an intrinsic connectivity networks in resting brain involved in attentional processes (Vincent et al., 2008; Markett et al., 2014). Using graph theory, a recent resting-state fMRI study also found EC was significantly correlated with regional efficiency of left thalamus (Xu et al., 2015). Cortico-subcortical circuits which connect the prefrontal cortex, the basal ganglia and the cerebellum via the thalamus are believed to serve as neuroanatomical substrates underlying EC (Heyder et al., 2004). The “dorsolateral prefrontal loop,” a circuit which contributed to the executive processing, involved projections from the dorsolateral prefrontal cortex to the lateral pallidum which in turn projects back to the dorsolateral prefrontal cortex via the thalamus (Alexander et al., 1986). Other “motor loop” or “oculomotor loop” also entailed the thalamus and putaman for information processing (Heyder et al., 2004). Significant BOLD activations within calcarine fissure were evoked during target stimuli to reveal the control mechanisms of attention (De Baene et al., 2015; Liu et al., 2016). From a structural network perspective, our finding of these areas thus provided further supports for their roles in information transfer underlying executive system. This suggest that task-free analysis of structural brain networks might help understand the neuroanatomical substrates of human behavior.

#### Alerting

For alerting component, we found significant correlations of nodal efficiency in the left thalamus. Previous fMRI studies have shown the functional activation in the thalamus during alerting (Fan et al., 2005; Schiff, 2008). A recent resting-state fMRI study also found that alerting was significantly correlated with regional efficiency of left thalamus (Xu et al., 2015). Thalamic and parietal regions were also activated during the rapid visual information processing, a task to test sustained attention (Lawrence et al., 2003). Using the positron emission tomography (PET) technique, regional cerebral bold flow increases were noted in the left thalamus during a vigilance task (Coull et al., 1996). Additionally, a recent cerebral MRI study showed the thalamus abnormality in Wilson’s Disease, which was typically affected by attention (Han et al., 2014). Thalamus, which connects with virtually every cortical area, is a good candidate to enhance alerting process. Our results of high regional efficiency in the thalamus indicated greater alerting performance, confirming its critical role for processing speed of alerting function.

The current study also found significant correlation between alerting and nodal efficiency of right inferior parietal lobule. Inferior parietal lobule, known as a region connected with visual and somatosensory cortices, has been involved in the perception of emotions and interpretation of sensory information (Radua et al., 2010). Also, it’s an important component of the fronto-parietal cognitive control system (Vincent et al., 2008) and implicated in sustained attention (Corbetta and Shulman, 2002). Furthermore, a recent fMRI study discovered significant associations between the centrality of the right inferior parietal lobule and right intraparietal on the one side and the alerting effect measured from ANT on the other (Markett et al., 2014). Based on structural connectivity, our study provided an anatomical evidence for inferior parietal being vital for information transferring implicated in alerting process.

We also found significant correlation between alerting and regional efficiency of middle temporal gyrus and pallidum. Middle temporal gyrus showed decreased BOLD activity during the rapid visual information processing task, which is used to assess sustained attention (Lawrence et al., 2003). Meditators, with better attentional performance to maintain focus on a particular object, showed less activity in middle temporal gyrus than non-meditators (Kozasa et al., 2012). ADHD brains showed decreased volume in the middle temporal gyrus (Carmona et al., 2005). Previous fMRI studies found abnormal activity ascribable to ADHD within the right pallidum (Di Martino et al., 2013; McLeod et al., 2014).

#### Orienting

A significant correlation was found between the orienting effect and paracentral lobule, inferior occipital gyrus and temporal pole (middle temporal gyrus). Paracentral lobule was identified as a member of the structural core in the structural brain networks from diffusion spectrum imaging data (Hagmann et al., 2008). A meta-analysis demonstrated the involvement of paracentral lobule in covert shifts of attention (Grosbras et al., 2005). Another meta-analysis found that the right paracentral lobule had a greater probability of activation in patients with ADHD than in controls (Dickstein et al., 2006). A resting-state fMRI study discovered that paracentral lobule showed significantly greater connectivity in late-life depression than non-depressed group (Kenny et al., 2010). Interestingly, the paracentral region was also activated during auditory attention shifting (Huang et al., 2012). Therefore, the paracentral lobule played important role in both structural and functional brain networks.

Inferior occipital cortex, served as a hub of the intrinsic fronto-parietal attentional network, also involved in shift of attention (Grosbras et al., 2005). Task-related fMRI revealed the activation for orienting in the lateral inferior occipital gyrus (Hietanen et al., 2006). An EEG study also revealed the key role played by the lateral occipital cortex in attentional modulations (Tallon-Baudry et al., 2005). Gitelman et al. (1999) concluded that the temporo-occipital activation, extending into the inferior temporal sulci was linked to the covert shifts of attention. Another fMRI study found that middle temporal gyrus exhibited greater activity when attention was shifted to vision than when it was shifted to audition (Shomstein and Yantis, 2004). Taken together, the paracentral lobule, as well as the inferior occipital gyrus could be the important anatomical substrates underlying the orienting function.

### Limitations

There are limitations in our study. Firstly, we employed deterministic tractography to define the structural network edges. Although this approach has been used in previous neuroimaging studies (Shu et al., 2012; Caeyenberghs et al., 2014), it might lose some existing fibers because of its limitation in tracking the crossing and long-distance fibers. Further studies based on advanced tractography methods (Zalesky and Fornito, 2009; Jeurissen et al., 2011) may identify more precise fiber pathways to solve this problem. Secondly, our study used the AAL-atlas to divide the human brain into 90 regions, which provides a macroscale view of brain networks. However, recent studies have shown that higher-spatial-resolution networks of up to 10240 parcels or of voxel-based scheme may provide an increased sensitivity to local properties (Hayasaka and Laurienti, 2010; Zalesky et al., 2010; Khundrakpam et al., 2015). Additional studies with higher-spatial-resolution networks are needed to validate our findings. Finally, the population in our study is within a narrow age range and in developing stage. Our findings may vary with time and be inconsistent with findings with children or adults. Further studies need to use wide age range to obtain more precise results.

## Conclusion

Using graph theory on structural brain networks from DTI data, this study investigated the relationships between the topological architecture and behavioral performances involved in attention. The findings of significant correlation of EC with global efficiency of the whole-brain structural networks indicated that the global efficiency of brain structural organization would provide a vital biological basis for the executive function. Furthermore, we observed specific brain regions related to alerting, orienting and EC components at the regional level, which provided evidences for understanding the fundamental structural substrates of attention function. These results suggest that graph theoretical analysis of structural brain networks could be an efficient way to reveal the underlying biological mechanism for cognitive functions.

## Author Contributions

MX, HG, BK, YL, WX, and SL designed the research. MX, HG, JX, GB, YL, LZ, YT, XG, SJ, WX, and AE performed the research. MX, BK, JX, GB, LZ, YT, XG, SJ, AE, and SL drafted and revised the paper. All authors contributed to discuss the results and have read and approved the final manuscript.

## Conflict of Interest Statement

The authors declare that the research was conducted in the absence of any commercial or financial relationships that could be construed as a potential conflict of interest.

## Footnotes

1 http://www.brainnetome.org/en/brat
